# TGF-β Activity Related to the Use of Collagen Membranes: In Vitro Bioassays

**DOI:** 10.3390/ijms21186636

**Published:** 2020-09-10

**Authors:** Layla Panahipour, Zahra Kargarpour, Bernadette Luza, Jung-Seok Lee, Reinhard Gruber

**Affiliations:** 1 Department of Oral Biology, School of Dentistry, Medical University of Vienna, 1090 Vienna, Austria; layla.panahipour@meduniwien.ac.at (L.P.); zahra.kargarpooresfahani@meduniwien.ac.at (Z.K.); bernadette.luza@gmx.at (B.L.); 2 Department of Periodontology, Research Institute for Periodontal Regeneration, College of Dentistry, Yonsei University, 03722 Seoul, Korea; cooldds@gmail.com; 3 Department of Periodontology, School of Dental Medicine, University of Bern, 3012 Bern, Switzerland; 4 Austrian Cluster for Tissue Regeneration, 1200 Vienna, Austria

**Keywords:** TGF-β activity, collagen membranes, guided bone regeneration, in vitro

## Abstract

Collagen membranes commonly used in guided bone regeneration are supposed to actively influence tissue regeneration and are not exclusively serving as passive barriers shielding away the soft tissue. The molecular mechanisms by which collagen membranes might affect tissue regeneration might involve the activation of transforming growth factor beta (TGF-β) signaling pathways. Here, we determined the TGF-β activity of supernatants and proteolytic lysates of five commercially available collagen membranes. The expression of TGF-β target genes interleukin 11 (IL11), NADPH oxidase 4 (NOX4), and proteoglycan 4 (PRG4) was evaluated by reverse transcriptase polymerase chain reaction and IL11 immunoassay in gingival fibroblasts. TGF-β signaling activation was further assessed by blocking the TGF-β receptor I kinase, a TGF-β neutralizing antibody, and showing the nuclear localization of phosphorylated Smad3 and total Smad2/3. We could identify two collagen membranes whose supernatants and lysates caused a robust increase of TGF-β receptor I kinase-dependent expression of IL11 in gingival fibroblasts. Moreover, the supernatant of a particular one membrane caused the nuclear localization of phosphorylated Smad3 and Smad2/3 in the fibroblasts. These results strengthen the evidence that some collagen membranes possess an intrinsic TGF-β activity that might actively influence the process of guided bone regeneration.

## 1. Introduction

Collagen membranes have been implemented in regenerative dentistry to stabilize and separate the bony defect from the soft tissue compartment during the early stages of regeneration [1]. The role of membranes in guided bone regeneration has been extended from the original concept of passively isolating bone-forming cells [1,2,3] to the current concept of actively influencing adjacent tissues [2,3]. Collagen membranes can be of xenogenic origin, for example, the porcine peritoneum or skin, following a decellularization and defatting process [4]. Similar to the extracellular matrix in the original tissues, collagen membranes of xenogenic origin are supposed to maintain part of the original biological activity after processing that goes beyond simply serving as barriers [2,3]. The impact of collagen membranes on the biological activity and consequently, the overall outcome of guided bone regeneration, however, remain enigmatic.

Collagen membranes from peritoneal membranes are rich in structural molecules, most of all COL1 as well as other members of the collagen family [5], also containing small leucine-rich repeat proteoglycan (SLRP) such as biglycan and decorin, exerting a brought spectrum of biological functions [6]. The extracellular matrix of the original tissue serves as a barrier, but it is the ability of collagens [7] but also SLRP [8] to bind growth factors that might maintain their biological functions after processing into collagen membranes. In vivo, cells populating the extracellular matrix are equipped with a panel of proteases that can release and also respond to the growth factors stored in an extracellular matrix [9]. It is possible that after processing the original extracellular matrix into collagen membranes that includes defatting, alkaline, and acid treatment, and dehydrated with acetone, at least part of this growth factors activity is maintained and ready to be released by cells infiltrating the matrix in guided bone regeneration (GBR).

Transforming growth factor beta (TGF-β) is among the candidate growth factors that is characteristically bound to collagen [7] and SLRP [10]. Once released, TGF-β may force fibroblast to produce even more collagen [11]. The role of TGF-β bound to collagen in vivo has not been fully understood. We can at least assume that collagen membranes used for GBR procedures contain TGF-β that remains its activity upon defatting, alkaline and acid treatment, and dehydration. So far, TGF-β was identified by immunoassay in the supernatant of collagen membranes from porcine peritoneum [12]. However, immunoassays identify TGF-β per se but not its activity—and it is the activity that might be impaired by the manufacturing process. Thus, if the TGF-β that is possibly stored in the membrane is also capable of activating the TGF-β signaling cascade in fibroblasts, has not been shown so far.

The overall aim of the present research is therefore to identify the TGF-β activity that is released from a panel of collagen membranes either spontaneous or upon their digestion with collagenase. We took advantage of our established bioassays allowing us to measure the TGF-β activity that is based on the expression of at least three sensitive target genes, namely IL11, NOX4, and PRG4 that were among the most sensitive genes in gene arrays [13,14,15]. Both IL11 and NOX4 are not only target genes, as there are also critically involved in mediating downstream TGF-β effects in cardiovascular and liver fibrosis [16,17] and systemic sclerosis [18]. Support for the TGF-β activity comes from blocking the TGF-β receptor 1 kinase with SB431542 [19] and the phosphorylation and the nuclear translocation of the canonical Smad2/3 signaling molecules [20]. This study evaluated the TGF-β activity on five commercially-available collagen membranes.

## 2. Material and Methods

### 2.1. Supernatants and Lysates of Collagen Membranes

Five different collagen membranes; Bio-Gide^®^ (BG), Fibro-Gide^®^ (FG), Mucograft^®^ (MG, all Geistlich Pharma AG, Wolhusen, Switzerland), Collagen Membrane-P (CMP; Genoss Co. Ltd.; Gwanggyo-ro, Yeongtong-gu, Suwon-si, Gyeonggi-do, Korea), Collagen Graft 2 (CG2; Genoss Co. Ltd.) were cut into rectangular pieces 15 mm × 10 mm. The membranes were then placed into 1 mL of serum-free DMEM (Sigma, St. Louis, MO, USA) for 72 h in a humidified atmosphere at 37 °C without shaking. Supernatants were collected with a pipette, and cells were immediately exposed to the undiluted solution. The other samples of collagen membranes were digested with 0.1% collagenase type 1 (Invitrogen Corporation, Grand Island, NY, USA) at room temperature with continuous shaking overnight followed by heating at 72 °C for 30 min. After centrifugation at 15,000 g for 2 min, the lysates were prepared to expose to cells immediately.

### 2.2. Primary Gingival Fibroblasts

Tissue samples of three independent donors were harvested from extracted third molars, and gingival fibroblasts were prepared after approval of the Ethical Committee of the Medical University of Vienna (EK Nr. 631/2007). Cells were cultured in a humidified atmosphere at 37 °C in growth medium consisting of DMEM, 10% fetal calf serum (Bio&Sell GmbH, Nuremberg, Germany) and 1% antibiotics (Sigma Aldrich, St. Louis, MO, USA). Cells were plated in growth medium at 30,000 cells/cm^2^ into culture dishes. The following day, fibroblasts were exposed to the supernatants and the lysates of the collagen membranes. SB431542, a TGF-β receptor I kinase inhibitor, was used at 10 µM (Calbiochem, Merck Millipore, Billerica, MA, USA). The TGF-β neutralizing pan-specific polyclonal rabbit IgG AB-100-NA (R&D Systems, Minneapolis, MN, USA) was used at 20 µg/mL. Cell conditioned medium was harvested after 24 h, centrifuged and stored frozen until subjected to immunoassay. Gene expression analysis and immunostainings were performed as indicated. Fibroblasts expanded for less than 10 passages were used for the experiments.

### 2.3. qRT-PCR Analysis and Immunoassay

Total RNA was isolated with the ExtractMe total RNA kit (Blirt S.A., Gdańsk, Poland). Reverse transcription was performed (LabQ, Labconsulting, Vienna, Austria). RT-PCR was done (LabQ, Labconsulting, Vienna, Austria) on a CFX Connect™ Real-Time PCR Detection System (Bio-Rad Laboratories, Hercules, CA). Primer sequences are hPRG4_F CAGTTGCAGGTGGCATCTC, hPRG4_R TCGTGATTCAGCAAGTTTCATC; hNOX4a_F TCTTGGCTTACCTCCGAGGA, hNOX4a_R CTCCTGGTTCTCCTGCTTGG; hGAPDH_F AAGCCACATCGCTCAGACAC, hGAPDH_R GCCCAATACGACCAAATCC. IL11 primer were from Bio-Rad (qHsaCEP0049951). The mRNA levels were calculated by normalizing to the housekeeping gene GAPDH using the ΔΔCt method after exponential expression transformation. For the immunoassay, the human IL11 and human TGF-β Quantikine ELISA kit was used (R&D Systems, Minneapolis, MN, USA). ELISA data was not normalized to an internal compound.

### 2.4. Immunofluorescence

Gingival fibroblasts were plated in growth medium onto Millicell^®^ EZ slides (Merck KGaA, Darmstadt, Germany). The following day, cells were switched to serum-free medium and either immediately exposed to the supernatant of the membrane for 30 min (Smad/2/3) or after an overnight starvation in serum-free medium (p-Smad3). Cells were then fixed in paraformaldehyde and blocked in 5% BSA and 0.3% Triton X in PBS at room temperature then permeabilization with 0.1% Triton X was performed. Following this, cells were incubated with p-Smad3 Ser423/425 (ab52903 rabbit, Abcam, Cambridge, UK) and with Smad2/3 antibody (D7G7 XP^®^ rabbit mAb #8685, Cell Signaling, MA, USA) for overnight at 4 °C. Following an Alexa Fluor^®^ 488-conjugated secondary antibody (1:1000; Anti-Rabbit, Cell signaling Technology, Danvers, MA, USA) was added for 1 h at room temperature. Cells were washed and mounted onto glass slides. Images were captured under a fluorescent microscope (Axio Imager M2, Carl Zeiss AG, Oberkochen, Germany).

### 2.5. Gelatin Zymography

Supernatant of the five membranes in Laemmli buffer were run on 10% SDS-PAGE gels containing 0.1% gelatin (Sigma). Gingival fibroblasts and Raw 264.7 condition medium were used as positive controls for MMP2 and MMP9. Following electrophoresis, gels were washed (50 mmol/L Tris HCl pH 7.5, 2.5% Triton X, 1 µM ZnCl_2_, 5 mmol/L CaCl_2_) two times for 30 min. Gels were then incubated for overnight in incubation buffer (50 mmol/L Tris HCl pH 7.5, 1% Triton X-100, 1 μM ZnCl_2_, 5 mmol/L CaCl_2_) at 37 °C. The next day, gels were stained for 30 min in Brilliant Blue R staining solution (Sigma) following destaining (methanol:acetic acid:water; 50:10:40). MMP2 and MMP9 appear as a clearance zone within the stained gel with a red band indicating 72 kDa.

### 2.6. Statistical Analysis

The experiments were repeated at least three times. Dot plot shows the median from all the experiments. Statistical analysis was based on the Friedman test and two-tailed Pair t-test. Analyses were performed using Prism v7 (GraphPad Software, La Jolla, CA, USA). Significance was set at *p* < 0.05.

## 3. Results

### 3.1. Supernatants of Two Collagen Membranes Increased TGF-β Target Genes

**Figure 1:**
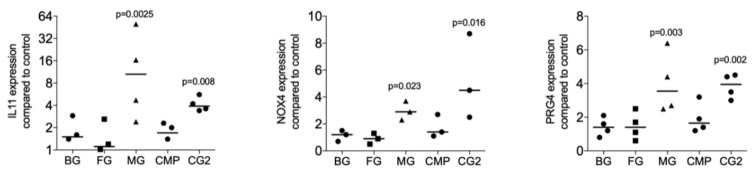
Supernatants of two collagen membranes increase TGF-β target genes.

To identify the possibility of the release of TGF-β by collagen membranes, five randomly selected collagen membranes were used for screening purposes. The fibroblasts being highly sensitive to TGF-β signaling were incubated with the supernatant of standardized pieces of collagen membranes. The TGF-β target genes IL11, NOX4, and PRG4 were the read-out of the bioassay as reported previously in the context of bone conditioned medium [15], acid bone lysates [21], and enamel matrix derivatives [14]. We found that among the five membranes tested, the MG and the CG2 provoked a significant increase expression of the IL11 (*p* = 0.0025 and *p* = 0.008), NOX4 (*p* = 0.023 and *p* = 0.016), and PRG4 (*p* = 0.003 and *p* = 0.002) based on gene expression analysis (Figure 1). Supernatants of BG, FG, and CMP failed to provoke a significant increase of the IL11 (*p* = 0.395, *p* = 0.450 and *p* = 0.508), NOX4 (*p* = 0.827, *p* = 0.827 and *p* = 0.190), and PRG4 (*p* = 0.395, *p* = 0.449 and *p* = 0.156). In support of the bioassays, traditional immunoassays detected active TGF-β1 in the supernatant of two independent preparations of MG (122 and 399 pg/mL) and CG2 (134 and 159 pg/mL). TGF-β1 in the supernatants of BG, FG, and CMP were not in the detection range of the assay.

Supernatants of collagen membranes Bio-Gide^®^ (BG), Fibro-Gide^®^ (FG), Mucograft^®^ (MG), Collagen Membrane-P (CMP), Collagen Graft 2 (CG2) were prepared by passive release into serum-free medium. Gingival fibroblasts were incubated with the supernatants of the collagen membranes. Reverse transcription PCR analysis for IL11, NOX4 and PRG4 was performed and the data are expressed as x-fold increase compared to unstimulated control cells. Statistical analysis was based on a Friedman test, based on non-parametric distribution of paired data with *p*-values indicated in the Figure. N = 3–4. 

### 3.2. Collagen Membranes Failed to Release MMP2 and MMP9 Activity

**Figure 2:**
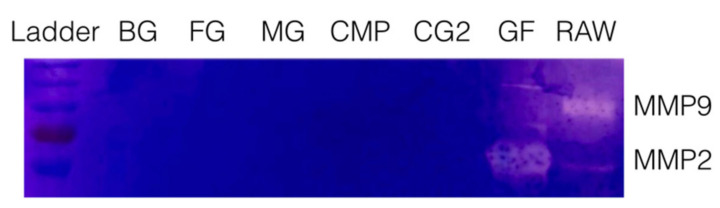
Zymography of the supernatant of collagen membranes.

To rule out that the release of TGF-β activity is associated with an intrinsic collagenase activity, a zymography of all supernatants was performed. Zymography revealed the expected bands showing the MMP2 and MMP9 proteolytic activity of the supernatants of gingival fibroblasts and Raw 264.7 macrophages (Figure 2). No bands indicating a proteolytic activity for gelatin were detected in the supernatants of collagen membranes. These results suggest that TGF-β activity of MG and CG2 is passively released and not liberated by collagenases of the collagen membranes.

Supernatants of collagen membranes Bio-Gide^®^ (BG), Fibro-Gide^®^ (FG), Mucograft^®^ (MG), Collagen Membrane-P (CMP), Collagen Graft 2 (CG2) were subjected to gelatin zymography. Neither of the collagen membranes but the supernatant of gingival fibroblasts (GF) and Raw 264.7 macrophages (RAW) caused a digestion of the substrate indicated by the bands taking up less dye.

### 3.3. Lysates of Two Collagen Membranes Increased TGF-β Target Genes

**Figure 3:**
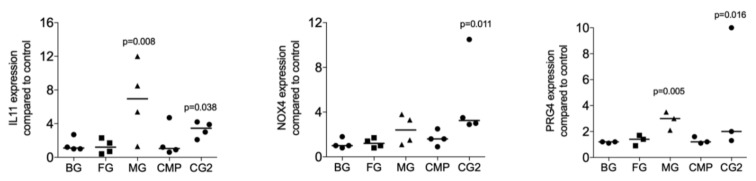
Lysates of Mucograft^®^ (MG) and Collagen Graft 2 (CG2) increase TGF-β target genes expression.

**Figure 4:**
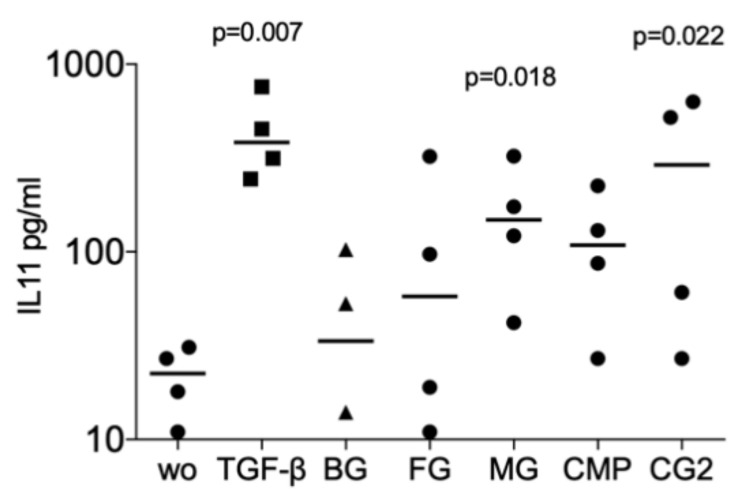
Supernatant and lysates of MG and CG2 increase interleukin 11 (IL11) signals in the immunoassay.

Next, we investigated whether collagenase digestion release TGF-β activity from the membranes that showed no positive signal of the supernatant. The results of collagenase digestion assays supported the observation gained by using the supernatants that MG (*p* = 0.008, *p* = 0.108 and *p* = 0.005), and CG2 (*p* = 0.038, *p* = 0.011 and *p* = 0.016), but not BG, FG and CMP increase the expression of the IL11 (*p* = 0.705, *p* = 0.850 and *p* = 0.705), NOX4 (*p* = 0.776, *p* = 0.570 and *p* = 0.220), and PRG4 (*p* = 0.326, *p* = 0.275 and *p* = 0.230) in gingival fibroblasts (Figure 3). Increased IL11 protein levels by supernatants and lysates of MG and the CG2 were also confirmed by immunoassay; again, it is the MG (*p* = 0.018), and CG2 (*p* = 0.002), but not BG, FG and CMP causing a significant increase in IL11 protein (Figure 4). These results indicate that collagenase digestion does change the TGF-β activity of membranes.

Lysates prepared by collagenase digestion of collagen membranes Bio-Gide^®^ (BG), Fibro-Gide^®^ (FG), Mucograft^®^ (MG), Collagen Membrane-P (CMP), Collagen Graft 2 (CG2) were used to stimulate gingival fibroblasts. Reverse transcription PCR analysis for IL11, NOX4 and PRG4 was performed, and the data are expressed as x-fold increase compared to unstimulated control cells. Statistical analysis was based on a Friedman test, based on non-parametric distribution of paired data. N = 3–5.

Supernatant and lysates prepared by the indicated collagenase digestion of collagen membranes Bio-Gide^®^ (BG), Fibro-Gide^®^ (FG), Mucograft^®^ (MG), Collagen Membrane-P (CMP), Collagen Graft 2 (CG2) were used to stimulate gingival fibroblasts. Immunoassay for IL11 was performed with the cell conditioned medium and the data are expressed as pg/mL. Statistical analysis was based on a Friedman test, based on non-parametric distribution of paired data. N = 3–4.

### 3.4. TGF-β Receptor I Kinase is Required to Increased TGF-β Target Genes

**Figure 5:**
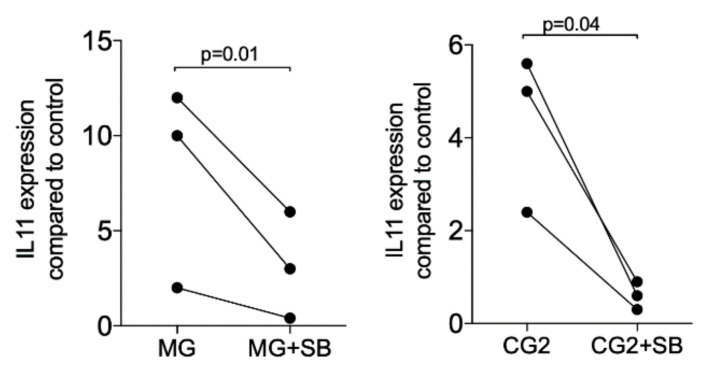
TGF-β receptor I kinase is required to increase IL11 in response to MG and CG2.

Upon the binding of TGF-β to its receptor, the signaling cascade is initiated [20]. Thus, we determined whether blocking of the TGF-β receptor 1 kinase with SB431542 affects the expression of IL11. In support of the notion that IL11 is a highly sensitive TGF-β target gene [13,14,15] and even mediates the effects of TGF-β in vivo [16,17], SB431542 completely blocked the expression when activated with the supernatant but also the lysates of MG and CG2 (Figure 5). Further support for the TGF-β activity in the supernatant of MG and CG2 comes from findings that the TGF-β neutralizing antibody reduced IL11 mRNA expression in two independent experiments from 19.0/18.3 to 3.2/1.4 (MG) and 2.0/28.3 to 0.7/0.9 (CG2). These results suggest that the increased expression of IL11 by supernatant of MG and CG2 requires TGF-β1 and the respective activation of the TGF-β receptor 1 kinase.

Gingival fibroblasts were exposed to the supernatant of Mucograft^®^ (MG) and Collagen Graft 2 (CG2) in the presence or absence of the TGF-β receptor 1 antagonist SB431542 (SB). Reverse transcription PCR analysis for IL11 was performed. Statistical analysis was based on a T-test of paired data. N = 3.

### 3.5. Smad2/3 Nuclear Translocation is Induced by Supernatants and Lysates of Two Collagen Membranes

**Figure 6:**
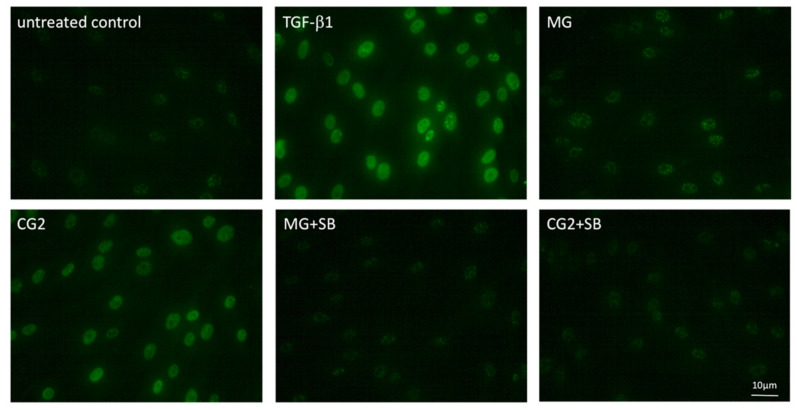
Phosphorylated smad3 nuclear translocation by MG and CG2 being blocked by SB431542.

**Figure 7:**
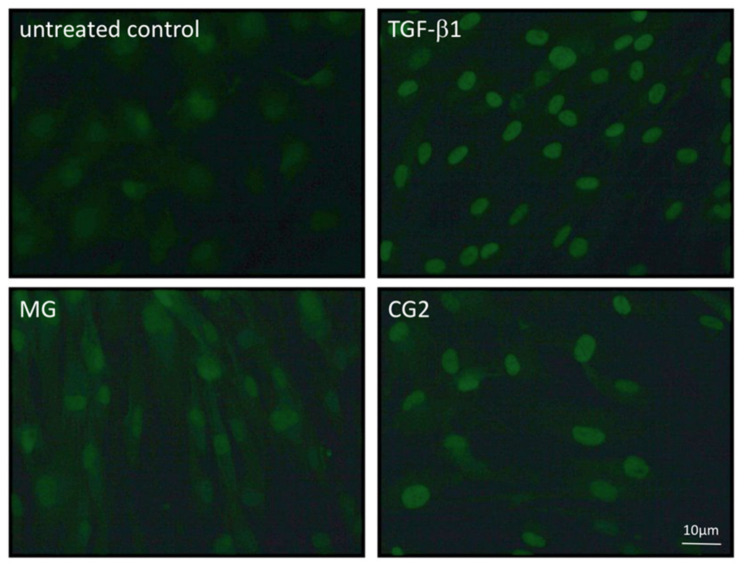
Smad2/3 nuclear translocation is induced by supernatants of MG and CG2.

Considering that activation of TGF-β receptor kinase leads to the phosphorylation and nuclear translocation of smad2/3 dimers an immunostaining was performed [20]. In support of the canonical TGF-β receptor signaling pathway, immunostainings revealed the nuclear staining of phosphorylated smad3, particularly with the supernatant of CG2 membranes (Figure 6). This translocation is reduced by the TGF-β receptor 1 antagonist SB431542 further supporting the conclusion that CG2 exert the activity via the release of TGF-β activity (Figure 6). Further support for the activation of TGF-β receptor signaling by supernatants of MG and CG2 comes from showing the nuclear translocation of total smad2/3 (Figure 7). Again, it was CG2 causing a clear accumulation of total smad2/3 in the nucleus. The nuclear translocation was less obvious with MG. Together, these results indicate that particular CG2 can activate the canonical TGF-β signaling cascade in gingival fibroblasts.

Gingival fibroblasts were exposed to the supernatant of Mucograft^®^ (MG) and Collagen Graft 2 (CG2), TGF-β (5 ng/mL) and SB431542 (SB; 10 µM) before an immunostaining was performed. Fluorescence signals show the presence of p-smad3 accumulating in the nucleus. Note that MG signal is weaker than p-smad3 staining observed with CG2.

Gingival fibroblasts were exposed to the supernatant and lysates of Mucograft^®^ (MG) and Collagen Graft 2 (CG2) before an immunostaining was performed. Nuclear staining indicates the presence of smad2/3 signaling proteins. Again, the MG signal is less sharp than the nuclear staining observed with CG2.

## 4. Discussion

Collagen membranes are used routinely in regenerative dentistry to shield the fast-growing soft tissue from the rather slow regenerating bone compartments. However, there is increasing evidence that the collagen membranes exceed their passive role as barriers. The collagen membranes are supposed to maintain part of their original function that includes serving as a pool of growth factors being released on demand by the local cells of the soft and hard tissue [2,3]. Based on this assumption and the identification of TGF-β released from collagen membranes by immunoassay reported here and elsewhere [12], we can support previous knowledge by showing that the supernatants of two out of five tested collagen membranes can initiate the canonical TGF-β signaling pathway that integrates the TGF-β receptor 1 kinase-dependent expression of IL11, NOX4 and PRG4, as well as phosphorylation and nuclear translocation of the Smad2/3 molecules. This basic research is important as we show that the processing of the original tissues, including the peritoneum and the skin by pH changes and defatting, followed by drying and sterilization, into collagen membranes allows maintaining at least part of the original TGF-β activity.

If we relate our findings to those of others, we like to acknowledge the pioneer work of Spinell et al. showing that porcine peritoneal collagen membranes release around 500 pg TGF-β1 / µl into the aqueous fraction within 72 h of incubation at room temperature [12], even though the data are not consistent with the respective thesis showing almost no TGF-β1 release (https://edoc.ub.uni-muenchen.de/25047/1/Saliter_Julia.pdf). We, however, could not confirm the TGF-β1 activity released from BG membranes and also the range of TGF-β1 released from MG and CG2 is in pg/mL, thus around 1000-time lower than in recent reports [12]. Moreover, and considering that the detection of TGF-β on the protein level not necessarily allows conclusions on the activity of the growth factor, we have performed a bioassay, refining previous observations to a functional level. This bioassay was used to report the TGF-β activity of bone conditioned medium [22], acid bone lysate [21], enamel matrix derivatives [14], and lysates of PRF membranes [13]. Surprisingly, TGF-β is not part of the proteomic signature of the MG membrane (Lee et al. unpublished) with maybe attributed to the detection limit of the method; it thus seems that a bioassay with oral fibroblasts being highly sensitive to TGF-β signaling is an appropriate cellular tool to identify this growth factor activity.

The clinical relevance of our analyses leaves room for speculations. The TGF-β activity released by MG and CG2 was passive, not involving cells. In vivo however, the target cells are equipped with a collagenase activity, that is basically all cells involved in wound healing and bone regeneration such as endothelial cells and their associated osteogenic progenitors, as well as macrophages, can produce matrix metalloproteinases, linking cell immigration and collagen degradation [23,24,25]. Cells are consequently capable of releasing and activating members of the TGF-β superfamily from the collagen matrix [26]. This is the process of how recombinant BMP-2 is released from the collagen matrix in a therapeutic application [9]. In support of this assumption, fibroblasts seeded onto collagen membranes previously loaded with TGF-β show the respective activation of TGF-β signaling [21]. To with extent, and if at all, the natural TGF-β activity intrinsic to MG and CG2 collagen membranes support the overall process of tissue regeneration and graft consolidation in a guided bone regeneration awaits further research. Nevertheless, considering that TGF-β is the most potent inducer of collagen synthesis and also IL11 [16,17] and NOX4 [18] are critically involved in fibrosis of various tissues, it can be speculated that collagen membranes support the formation of new extracellular matrix. Activation of IL11 [16,17] and NOX4 [18] but also the increased production of collagen by immigrating fibroblasts might seem important at sites of soft tissue augmentation when the replacement of the temporary collagen membrane with a new extracellular matrix is the goal.

The present study has limitations that should be considered as an inspiration for future research. First, our findings that two out of the five membranes tested contain and release TGF-β activity is restricted to an in vitro bioassay; hence we have no data supporting that this observation translates into the formation on new extracellular matrix in vivo. Second, we cannot rule out that also the other three membranes are rich in TGF-β but the lack of activity is due to the inactivation during processing of the original tissue. Our research might inspire future studies to identify the steps in the processing of the collagen membranes that maintain or abolish the activity of growth factors such as TGF-β. Third, we have worked with supernatants of collagen membranes, so we cannot rule out that cells that are in intimate contact with the collagen membrane might provoke a TGF-β response; also, in the three membranes that failed to release detectable TGF-β activity. Nevertheless, this scenario is rather unlikely as we have digested the membranes with collagenase and the supernatant failed to induce IL11, NOX4 and PRG4 expression by fibroblasts. Fourth, we have no explanation so far why it is particular the CG2, and only to a lesser extent, the MG membranes that caused a clear nuclear translocation of the p-smad3 and smad2/3, even though with respect to gene expression and immunoassays, they behaved rather similar. Finally, we have not considered the batch-to-batch variation of the collagen membranes.

In conclusion, our data are one step towards deciphering the biological activity of commercially available collagen membranes, as we have identified at least two out of five membranes that considerably release a TGF-β activity detectable by an in vitro bioassay.
